# Supplementary material for: Unbroken 𝒫𝒯-symmetry in the absence of gain or loss
Source: Nat Commun. 2025 Sep 5;16:8225. doi: 10.1038/s41467-025-63242-3 (PMC12413444; doi:10.1038/s41467-025-63242-3)
Supplement: Supplementary file 1 — Supplementary Information [file 41467_2025_63242_MOESM1_ESM.pdf]

Unbroken $\mathcal{PT}$-symmetry in the absence of gain or loss: Supplementary information

Johannes Bentzien[1], Julien Pinske[2], Lukas J. Maczewsky[1], Steffen Weimann[1], Matthias Heinrich[1], Stefan Scheel[1] & Alexander Szameit[1]*

[1] Institute of Physics, University of Rostock, Albert-Einstein-Str. 23, 18059 Rostock, Germany. [2] Niels Bohr Institute, University of Copenhagen, Blegdamsvej 17, DK-2100 Copenhagen, Denmark. * alexander.szameit@uni-rostock.de

Contents

Supplementary Note 1: Tight-binding approximation (Coupled-mode theory) 2

Supplementary Note 2: Normal mode expansion 4

Supplementary Note 3: Classification 8

Supplementary Note 4: Hermiticity and orthogonality of coupled modes 9

Supplementary Note 5: Orthogonal vs. nonorthogonal modes in the directional coupler 10

Supplementary Note 6: NOCMT of the three-waveguide coupler 11

Supplementary Note 7: NOCMT for more than three waveguides 13

Supplementary Note 8: Results of all measurement sets 14

Supplementary Note 9: Eigenvalues and -frequencies with and without detuning 16

Supplementary Note 10: Fluorescence microscopy 19

Supplementary References 21

Supplementary Note 1: Tight-binding approximation (Coupled-mode theory)

The evolution of light in a system of parallel waveguides is governed by the paraxial Helmholtz equation

$$\mathrm{i}\partial_z E(x,y,z) = -\left(\frac{1}{2n_0k_0}\left[\partial_x^2 + \partial_y^2\right] + k_0\Delta n(x,y)\right) E(x,y,z), \tag{S1}$$

which is mathematically similar to the time-dependent Schrödinger equation. It can be derived directly from the wave equation only by assuming paraxiality[1], i.e. by neglecting the second-order derivative in propagation direction. In this vein, the propagation direction z acts as the time coordinate t of a quantum-mechanical system. Consequently, the physics of quantum mechanics can be emulated by classical, paraxial systems such as photonic waveguide lattices[2]. In arrays of coupled waveguides, light remains bound by the refractive index landscape and therefore typically remains inside the waveguide cores – similar to the confinement of the electronic wave function of electrons in the Coulomb potentials of the atoms in a crystalline lattice.

Along these lines, the tight-binding approximation from solid state physics[3] can be readily applied by expanding the propagating wave function into modes of the single waveguides, each further separated into transverse *waveguide modes* $w_k(x,y)$ (also known as transverse *mode fields*), longitudinal modal *amplitudes* $a_k(z)$ and a wave number β_k

$$E(x,y,z) = \sum_{k=1}^{N} a_k(z) w_k(x,y) \mathrm{e}^{-\mathrm{i}\beta_k z}. \tag{S2}$$

Assuming symmetric waveguide shapes, the transverse mode profiles should be localized parity-symmetrically around their respective waveguide and thus they are defined via fulfilling their own Helmholtz equation for the refractive index profile of a hypothetical isolated waveguide[1]

$$0 = \left(\frac{1}{2n_0k_0}\left[\partial_x^2 + \partial_y^2\right] + k_0\Delta n_k(x,y)\right) w_k(x,y).$$

The final discretized set of equations can be obtained by inserting Eq. (S2) into Eq. (S1). The evanescent coupling coefficients are defined via

$$c_{jk} = k_0 \sum_{m\neq j} \int \Delta n_m w_k^* w_j \mathrm{d}x\mathrm{d}y, \tag{S3}$$

where the diagonal elements are the self-coupling $\delta_k = c_{kk}$, and the mode overlap is the scalar product

$$\kappa_{jk} = \int w_k^* w_j \mathrm{d}x\mathrm{d}y.$$

In the end, only the modal amplitudes $a_k(z)$ contain the light propagation of each waveguide. The transverse waveguide modes in return determine the systems' parameters, which determine the dynamics of the system. In this sense, the coupled-mode theory (CMT) for optical waveguides can be developed by transforming the continuous system described by the paraxial Helmholtz equation to a discrete system described by a discrete Schrödinger equation[4]

$$\mathrm{i}P\partial_z \mathbf{a} = -K\mathbf{a}, \tag{S4}$$

also known as coupled-mode equations, where the Hamiltonian $H = P^{-1}K$ with $K = C + PB$ contains the corresponding constants. The real-valued matrices defining the Hamiltonian are the power matrix $P = \left(\kappa_{jk}\right)_{jk}$, the coupling matrix $C = \left(c_{jk}\right)_{jk}$ and the matrix $B = \mathrm{diag}((\beta_k)_k)$ containing the propagation constants. It follows that, if P and K are Hermitian (i.e. real symmetric) and do not commute, i.e. $[K, P] \neq 0$ and $[K, P^{-1}] \neq 0$, it is

$$H^\dagger = (P^{-1}K)^\dagger = K^\dagger(P^{-1})^\dagger = KP^{-1} \neq H,$$

which means the real-valued non-symmetric matrix H is necessarily non-Hermitian. It is further pseudo-Hermitian[5] as it fulfills

$$H^\dagger = KP^{-1}KK^{-1} = KHK^{-1}.$$

A system of non-overlapping modes is equivalent to orthogonality of the system, i.e., $\int w_k^* w_j \mathrm{d}x\mathrm{d}y = \delta_{jk}$ (Kronecker delta) or $P = \mathbb{I}_{N\times N}$. This is the case when the eigenmode (also known as supermode) of the ground state (cf. Supplementary Fig. 10a) is approaching zero in the inter-waveguide regions. If these conditions are sufficiently well fulfilled, orthogonal coupled-mode theory (OCMT) can be employed. The more general case without assuming orthogonality is called nonorthogonal coupled-mode theory (NOCMT).

Supplementary Note 2: Normal mode expansion

Analogously to the tight-binding mode expansion Eq. (S2), one can expand the electric field in transverse *normal modes* $v_k(x,y)$ (Supplementary Fig. 1d-f) and its corresponding longitudinal modal *amplitudes* $b_k(z)$

$$E(x,y,z) = \sum_{k=1}^{N} b_k(z) v_k(x,y) \mathrm{e}^{-\mathrm{i}\beta_k z}.$$

The difference between both expansions is that the transverse normal modes, contrarily to the transverse waveguide modes, are per definition always orthogonal ($\int v_k^* v_j \mathrm{d}x\mathrm{d}y = \delta_{jk}$), and thus can be constructed from the systems' supermodes (Supplementary Fig. 1a-c). As a result, their amplitudes $b_k(z)$ are not directly accessible via an experiment, in contrast to the waveguide mode amplitudes $a_k(z)$, that can be measured by means of the local fluorescence efficiency η_k (Supplementary Fig. 1g-i).

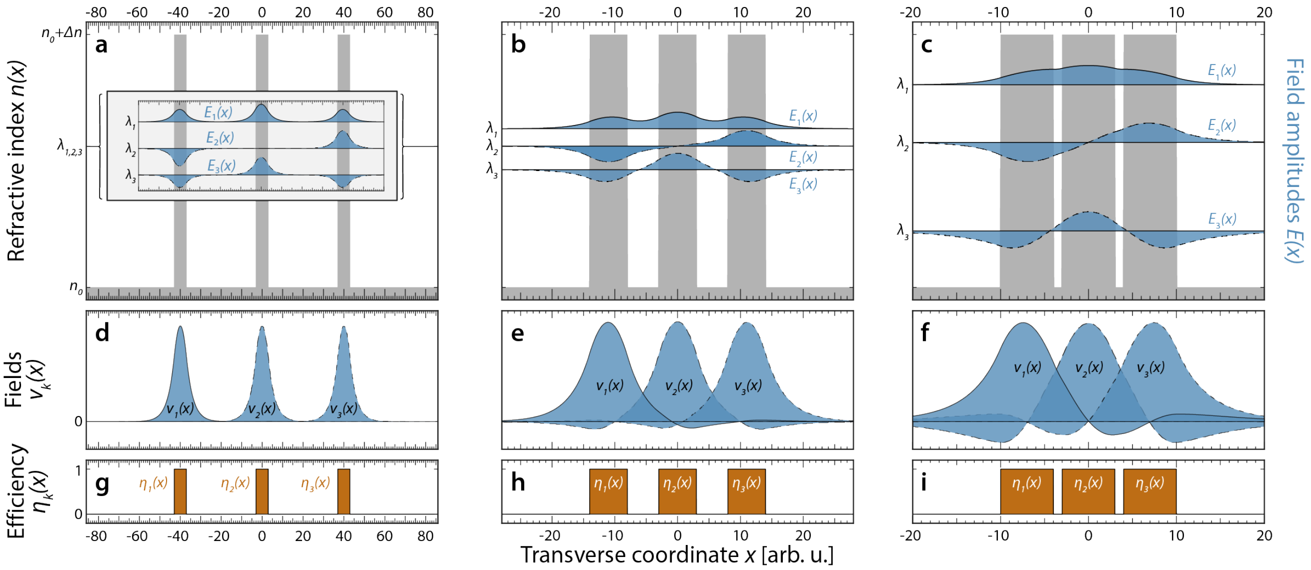

Supplementary Figure 1 | Projective non-Hermiticity in coupled-mode theory and fluorescence measurements (part 1). (cf. main text Fig. 2a-i) (**a-c**) Refractive index landscape (grey) of three identical step-index waveguides and resulting set of supermodes $E_k(x)$ for different center-to-center waveguide spacings $d = 40$ arb. u. (**a**), $d = 11$ arb. u. (**b**) and $d = 7$ arb. u. (**c**). The vertical position of the supermodes indicates their respective eigenvalues λ_k. (**d-f**) *Normal modes* $v_k(x)$ constructed from superpositions of the supermodes $E_k(x)$. For large waveguide separations (**d**), the normal modes $v_k(x)$ become indistinguishable from the parity-symmetric modes $w_k(x)$ of hypothetical isolated waveguides at the same position. For smaller waveguide separations (**e,f**), $v_k(x)$ and $w_k(x)$ diverge as the normal modes incur contributions from the other waveguides as well. While normal modes are per definition always orthogonal, the nonorthogonality of the system is embodied by the waveguide modes. (**g-i**) As simplified model for the waveguide fluorescence mechanism, the fluorescence efficiency $\eta_k(x)$ can be assumed to be identical to the index profile, i.e. uniform inside and zero outside the guides.

These two different modal amplitudes are connected via $\mathbf{b} = Q\mathbf{a}$, where the transformation Q factorizes the power matrix via $P = Q^{\dagger}Q$. Because the matrix Q is invertible our nonorthogonal projective method is reversible. For the general power matrix P in our three-waveguide system

$$P = \begin{pmatrix} 1 & \kappa_{12} & \kappa_{13} \\ \kappa_{12}^{*} & 1 & \kappa_{23} \\ \kappa_{13}^{*} & \kappa_{23}^{*} & 1 \end{pmatrix},$$

the matrix Q can have the following form

$$Q = \begin{pmatrix} 1 & \kappa_{12} & \kappa_{13} \\ 0 & \sqrt{1-|\kappa_{12}|^2} & \frac{\kappa_{23}-\kappa_{12}^{*}\kappa_{13}}{\sqrt{1-|\kappa_{12}|^2}} \\ 0 & 0 & \sqrt{1-|\kappa_{13}|^2-\frac{|\kappa_{23}-\kappa_{12}^{*}\kappa_{13}|^2}{1-|\kappa_{12}|^2}} \end{pmatrix}.$$

However, this matrix Q is not unique and can be modified by any unitary matrix U because

$$\tilde{P} = \tilde{Q}^{\dagger}\tilde{Q} = (UQ)^{\dagger}UQ = Q^{\dagger}U^{\dagger}UQ = Q^{\dagger}Q = P.$$

The mechanism of measuring $|a_k|^2$ is explained in the Supplementary Figs. 2, 3, and 4 for the three supermodes $E_k(x,y)$. The individual on-site intensities $|a_k|^2$ are proportional to the overlap between the local fluorescence efficiency η_k and the propagating mode ($\int \eta_k |E(z)|^2 \mathrm{d}x\mathrm{d}y \propto |a_k(z)|^2$, cf. orange areas Supplementary Figs. 2a-c, 3a-c and 4a-c and orange bars in Supplementary Figs. 2d-f, 3d-f and 4d-f). The proportionality factor is the total fluorescence efficiency of the orthogonal case. To calculate $|b_k|^2$, there is a simple connection between the electric field, the transverse normal modes and its modal amplitudes. The overlap between the propagating mode $E(x,y,z)$ and the normal mode $v_k(x,y)$ gives rise to the corresponding amplitude $b_k(z)$, because it is

$$\begin{aligned} \left|\int v_k^{*}(x,y)E(x,y,z)\mathrm{d}x\mathrm{d}y\right|^2 &= \left|\int v_k^{*}(x,y)\sum_{j=1}^{N} b_j(z)v_j(x,y)\mathrm{e}^{-\mathrm{i}\beta_j z}\,\mathrm{d}x\mathrm{d}y\right|^2 \\ &= \left|\sum_{j=1}^{N} b_j(z)\int v_k^{*}(x,y)v_j(x,y)\mathrm{d}x\mathrm{d}y\,\mathrm{e}^{-\mathrm{i}\beta_j z}\right|^2 \\ &= \left|\sum_{j=1}^{N} b_j(z)\delta_{kj}\,\mathrm{e}^{-\mathrm{i}\beta_j z}\right|^2 = \left|b_k(z)\mathrm{e}^{-\mathrm{i}\beta_k z}\right|^2 = |b_k(z)|^2. \end{aligned}$$

The results of $|b_k|^2$ for the three supermodes can be seen at the blue bars in Supplementary Figs. 2d-f, 3d-f and 4d-f.

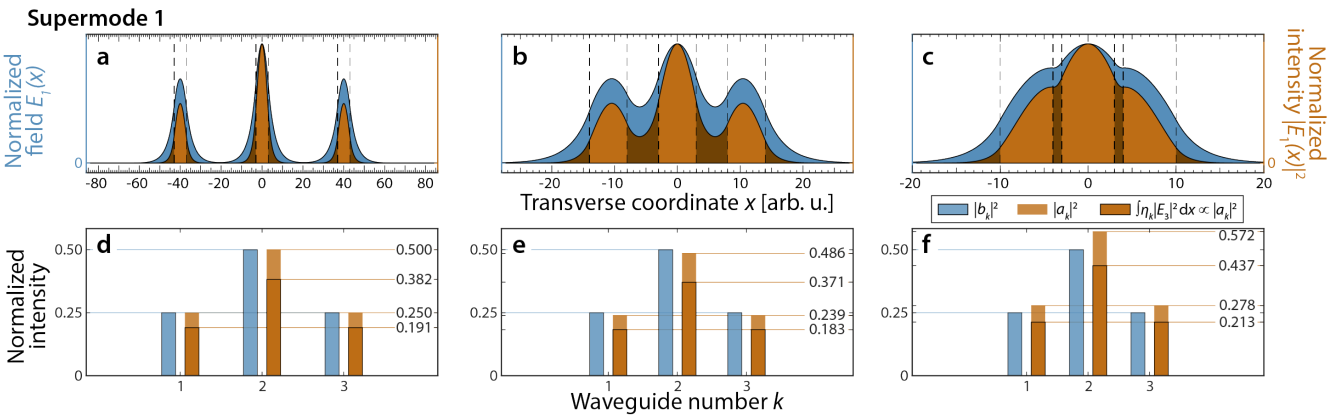

Supplementary Figure 2 | Projective non-Hermiticity in coupled-mode theory and fluorescence measurements (part 2). (**a-c**) As shown here for the example of the first supermode $E_1(x)$, fluorescence imaging does not measure the *overall* intensity distribution (dark orange) but rather the *on-site* fraction of it (orange). (**d-f**) In contrast to the normal mode occupations $(|b_k|^2)_k = \left(\frac{1}{4}, \frac{1}{2}, \frac{1}{4}\right)$ (blue bars), the on-site intensities $|a_k|^2$ (orange bars) are proportional to the overlap with the fluorescent core regions and therefore may dynamically vary upon propagation as in general $\sum_k |a_k|^2 \neq 1$. The proportionality factor is the total fluorescence efficiency of the orthogonal case. In the orthogonal regime (left column), a_k and b_k are identical as $w_k(x)$ and $v_k(x)$ become indistinguishable.

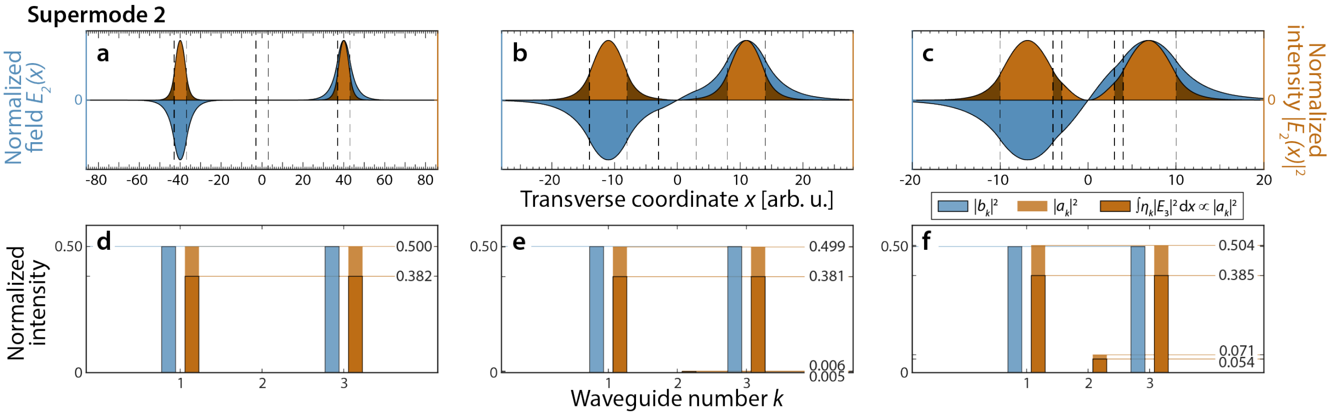

Supplementary Figure 3 | Projective non-Hermiticity in coupled-mode theory and fluorescence measurements (part 3). (**a-c**) As shown here for the example of the second supermode $E_2(x)$, fluorescence imaging does not measure the *overall* intensity distribution (dark orange) but rather the *on-site* fraction of it (orange). (**d-f**) In contrast to the normal mode occupations $(|b_k|^2)_k = \left(\frac{1}{2}, 0, \frac{1}{2}\right)$ (blue bars), the on-site intensities $|a_k|^2$ (orange bars) are proportional to the overlap with the fluorescent core regions and therefore may dynamically vary upon propagation as in general $\sum_k |a_k|^2 \neq 1$. The proportionality factor is the total fluorescence efficiency of the orthogonal case. In the orthogonal regime (left column), a_k and b_k are identical as $w_k(x)$ and $v_k(x)$ become indistinguishable.

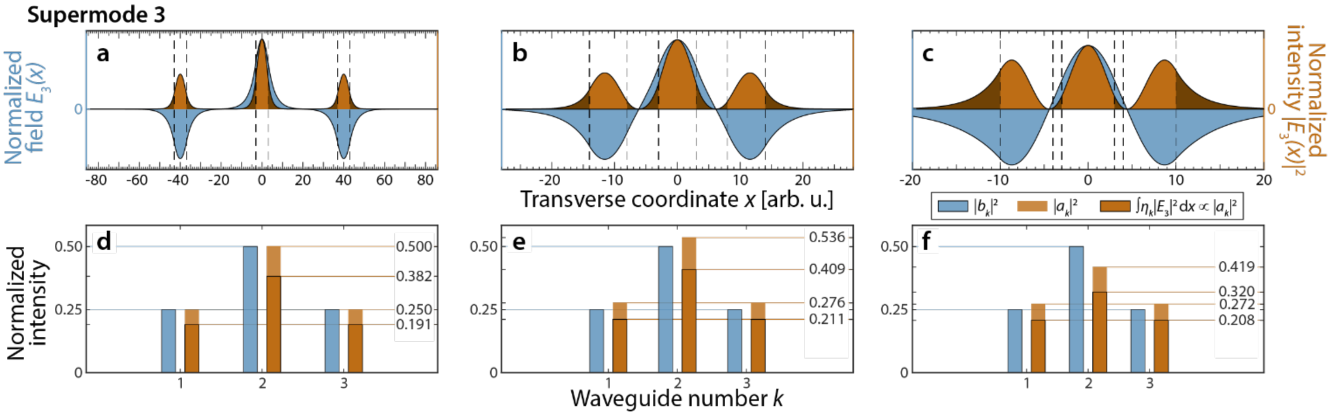

Supplementary Figure 4 | Projective non-Hermiticity in coupled-mode theory and fluorescence measurements (part 4). (cf. main text Fig. 2j-o) (**a-c**) As shown here for the example of the third supermode $E_3(x)$, fluorescence imaging does not measure the *overall* intensity distribution (dark orange) but rather the *on-site* fraction of it (orange). (**d-f**) In contrast to the normal mode occupations $(|b_k|^2)_k = \left(\frac{1}{4}, \frac{1}{2}, \frac{1}{4}\right)$ (blue bars), the on-site intensities $|a_k|^2$ (orange bars) are proportional to the overlap with the fluorescent core regions and therefore may dynamically vary upon propagation as in general $\sum_k |a_k|^2 \neq 1$. The proportionality factor is the total fluorescence efficiency of the orthogonal case. In the orthogonal regime (left column), a_k and b_k are identical as $w_k(x)$ and $v_k(x)$ become indistinguishable.

Supplementary Note 3: Classification

The Venn diagram in Supplementary Fig. 5 illustrates how the nonorthogonal coupled-mode theory (NOCMT) and $\mathcal{PT}$-symmetric systems are embedded into the space of all complex $N \times N$-matrices.

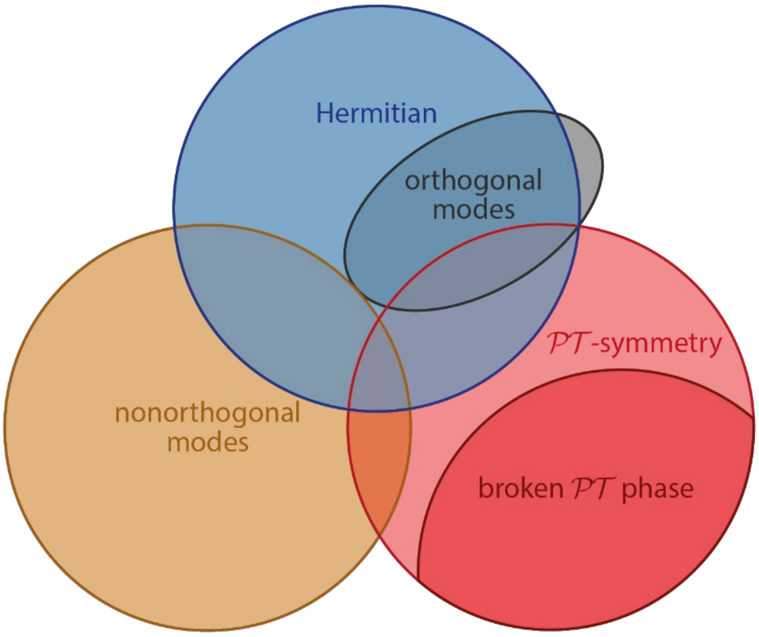

Supplementary Figure 5 | Venn diagram containing subsets of complex $N \times N$-matrices. Real-symmetric matrices consist of systems with orthogonal modes or those that have vanishing commutator $[P, K] = 0$. If P and K do not commute, the NOCMT yields a non-Hermitian matrix. The latter can possess $\mathcal{PT}$-symmetry but is always restricted to an unbroken $\mathcal{PT}$ phase. In contrast, gain-loss systems can possess real or complex spectrum thus reaching the area of broken $\mathcal{PT}$-symmetry.

NOCMT leads to a Hermitian system if P and K commute or, more specifically, when the modes become orthogonal by virtue of a sufficient spacing between the individual waveguides.

Supplementary Note 4: Hermiticity and orthogonality of coupled modes

An interesting note is that in our case (non)orthogonality and (non-)Hermiticity coincides, which is due to the common assumption of symmetric coupling. However, the definition according to Eq. (S3) in general would allow for asymmetric couplings, which would yield waveguide systems with orthogonal modes can have a non-Hermitian light distribution as well. Even the directional coupler may in general be non-Hermitian since

$$c_{12} = k_0 \int \Delta n_2 w_2^* w_1 \mathrm{d}x\mathrm{d}y \neq c_{21} = k_0 \int \Delta n_1 w_1^* w_2 \mathrm{d}x\mathrm{d}y,$$

for example, when a detuning is introduced to the system ($\Delta n_1 \neq \Delta n_2$). We were able to observe this effect in the directional coupler at small distances by looking at the total intensity distribution measured by the fluorescence microscopy. The result for a separation of 9 µm is seen in Supplementary Fig. 6a, which is similar to Fig. 4a,b in the main text but for the directional coupler. The sum of the measured intensity distributions of the two waveguides reveals a sinusoidal oscillation with double the frequency of the single waveguides. The variable total intensity is a result of the non-Hermiticity due to the non-symmetric coupling. This effect diminishes for larger separations. However, the OCMT can still be applied in this directional coupler assuming symmetric coupling to a good approximation as the exponential behavior for the coupling constant is still obtained (see Supplementary Fig. 6b). Nonetheless, this experiment shows that the assumption of symmetric coupling should be treated with caution.

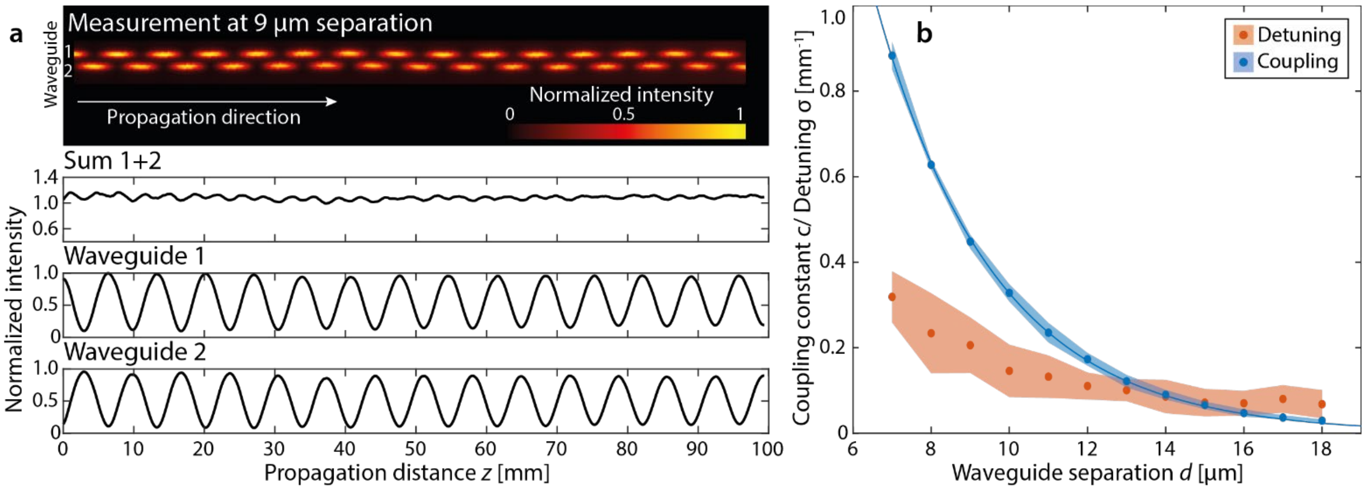

Supplementary Figure 6 | Results of measurement and simulation for the directional coupler. (**a**) Experimental result of the fluorescence measurement at 9 µm waveguide separation. (**b**) Evaluation of the coupling and detuning constant dependent on the waveguide separation from 7 to 18 µm determined from the fluorescence measurements.

Supplementary Note 5: Orthogonal vs. nonorthogonal modes in the directional coupler

The simplest waveguide array on which the coupled-mode theory can be applied is the directional coupler. To achieve $\mathcal{PT}$-symmetry, the index profile has to be symmetric, i.e. there is no detuning. For the symmetric coupling, the matrices C, B and P are

$$C = \begin{pmatrix} \delta & c \\ c & \delta \end{pmatrix}, \qquad B = \begin{pmatrix} \beta & 0 \\ 0 & \beta \end{pmatrix}, \qquad P = \begin{pmatrix} 1 & \kappa \\ \kappa & 1 \end{pmatrix}.$$

In the orthogonal coupled-mode theory (OCMT), the Hamiltonian is simply

$$H = C + B = \begin{pmatrix} \delta + \beta & c \\ c & \delta + \beta \end{pmatrix}.$$

In the more general nonorthogonal coupled-mode theory (NOCMT), the Hamiltonian follows as

$$H = P^{-1}C + B = \begin{pmatrix} \frac{\delta - c\kappa}{1-\kappa^2} + \beta & \frac{c - \delta\kappa}{1-\kappa^2} \\ \frac{c - \delta\kappa}{1-\kappa^2} & \frac{\delta - c\kappa}{1-\kappa^2} + \beta \end{pmatrix} = \begin{pmatrix} \delta' + \beta & c' \\ c' & \delta' + \beta \end{pmatrix}.$$

Both Hamiltonians are mathematically identical (especially when defining $\delta' = \frac{\delta - c\kappa}{1-\kappa^2}$ and $c' = \frac{c - \delta\kappa}{1-\kappa^2}$) and hence result in the same intensity distributions. The overlap parameter κ cannot be measured individually because it is hidden in the effective coupling constant c'. Thus, the directional coupler is not suitable to measure nonorthogonal modes.

Supplementary Note 6: NOCMT of the three-waveguide coupler

The dynamics of the three-waveguide coupler is governed by the discrete Schrödinger equation (S4). In the NOCMT regime, the overlap κ_{jk} can no longer be neglected and the Hamiltonian $H = P^{-1}C + B$ is composed of the matrices C, B and P. In the case of different refractive index contrasts of the waveguides (e.g. induced by stress fields) the propagation constants β_k are not equal and the matrix B can therefore no longer be neglected either. Assuming symmetric coupling (for simplicity and because its exponential behavior is still obtained), equal outer waveguides (for $\mathcal{PT}$-symmetry) and neglecting higher-order couplings (because of the exponential dependence on the distance[6]), the matrices C, B and P look as follows

$$C = \begin{pmatrix} \delta_1 & c & 0 \\ c & \delta_2 & c \\ 0 & c & \delta_1 \end{pmatrix}, \qquad B = \begin{pmatrix} \beta_1 & 0 & 0 \\ 0 & \beta_2 & 0 \\ 0 & 0 & \beta_1 \end{pmatrix}, \qquad P = \begin{pmatrix} 1 & \kappa & 0 \\ \kappa & 1 & \kappa \\ 0 & \kappa & 1 \end{pmatrix}.$$

The Hamiltonian can be simplified by converting the reference frame from a_k to A_k in the form

$$\mathbf{A} = \mathbf{a} \exp\left[\mathrm{i}\left(\beta_1 + \frac{\delta_1(1-\kappa^2) - c\kappa}{1-2\kappa^2}\right) z\right]$$

and by introducing an effective coupling constant $\tilde{c}$, the total detuning σ and the self-coupling induced detuning Δ as follows

$$\tilde{c} = \frac{c - \delta_1\kappa}{1-2\kappa^2}, \qquad \sigma = \beta_2 - \beta_1 + \frac{\delta_2 - \delta_1}{1-2\kappa^2}, \qquad \Delta = \frac{\delta_1 - \delta_2}{1-2\kappa^2}.$$

In the new reference frame, the Hamiltonian reads

$$H = \begin{pmatrix} 0 & \tilde{c} + \kappa\Delta & -\kappa\tilde{c} \\ \tilde{c} & \sigma - \kappa\tilde{c} & \tilde{c} \\ -\kappa\tilde{c} & \tilde{c} + \kappa\Delta & 0 \end{pmatrix}. \qquad \text{(S5)}$$

Solving the discrete Schrödinger equation (S4) with this Hamiltonian for the arbitrary initial condition

$$A_1(0) = \sqrt{P_1}, \qquad A_2(0) = \sqrt{P_2}, \qquad A_3(0) = \sqrt{P_3}$$

with $P_1 + P_2 + P_3 = 1$ yields the following result for the intensity distribution via $I_k(z) \propto |a_k(z)|^2 = |A_k(z)|^2$

$$I_1(z) \propto \left(\frac{\sqrt{P_1} - \sqrt{P_3}}{2}\right)^2 + \left(\frac{\sqrt{P_1} + \sqrt{P_3}}{2}\right)^2 \cos^2(\omega_1 z) + \frac{k_1^2}{\omega_1^2}\sin^2(\omega_1 z)$$
$$+ \frac{P_1 - P_3}{2}\cos(\omega_1 z)\cos(\omega_2 z) + \left(\sqrt{P_1} - \sqrt{P_3}\right)\frac{k_1}{\omega_1}\sin(\omega_1 z)\sin(\omega_2 z),$$

$$I_2(z) \propto P_2\cos^2(\omega_1 z) + \frac{k_2^2}{\omega_1^2}\sin^2(\omega_1 z), \qquad \text{(S6)}$$

$$I_3(z) \propto \left(\frac{\sqrt{P_1} - \sqrt{P_3}}{2}\right)^2 + \left(\frac{\sqrt{P_1} + \sqrt{P_3}}{2}\right)^2 \cos^2(\omega_1 z) + \frac{k_1^2}{\omega_1^2}\sin^2(\omega_1 z)$$
$$- \frac{P_1 - P_3}{2}\cos(\omega_1 z)\cos(\omega_2 z) - \left(\sqrt{P_1} - \sqrt{P_3}\right)\frac{k_1}{\omega_1}\sin(\omega_1 z)\sin(\omega_2 z),$$

with the constants

$$\omega_1 = \sqrt{2\tilde{c}^2 + \frac{\sigma^2}{4} + 2\tilde{c}\kappa\Delta}, \tag{S7}$$

$$\omega_2 = \frac{4\kappa\tilde{c} - \sigma}{2}, \tag{S8}$$

$$k_1 = \sqrt{P_2}(\tilde{c} + \kappa\Delta) - \left(\sqrt{P_1} + \sqrt{P_3}\right)\frac{\sigma}{4}, \tag{S9}$$

$$k_2 = \left(\sqrt{P_1} + \sqrt{P_3}\right)\tilde{c} + \sqrt{P_2}\frac{\sigma}{2}. \tag{S10}$$

In the experiment, the analytical solutions from Eq. (S6) can be fitted to the extracted individual intensity distributions from the fluorescence measurement (see Fig. 4a,b in the main text). The received fit parameters ω_1, ω_2, k_1 and k_2 can be used to get the physical parameters $\tilde{c}$, κ, σ and Δ, because the system of equations (S7)-(S10) is analytically solvable:

$$\tilde{c} = \frac{k_2^2 - P_2\omega_1^2}{k_2\left(\sqrt{P_1} + \sqrt{P_3}\right) - 2k_1\sqrt{P_2}},$$

$$\sigma = 2\tilde{c}\,\frac{\left(\sqrt{P_1} + \sqrt{P_3}\right)\sqrt{P_2}\,\omega_1^2 - 2k_1k_2}{k_2^2 - P_2\omega_1^2},$$

$$\kappa = \frac{\sigma + 2\omega_2}{4\tilde{c}},$$

$$\Delta = \frac{\tilde{c}}{\kappa}\left(\frac{\left(\sqrt{P_1} + \sqrt{P_3}\right)^2\omega_1^2 - 4k_1^2}{2\left(k_2^2 - P_2\omega_1^2\right)} - 1\right).$$

Supplementary Note 7: NOCMT for more than three waveguides

Our method can easily be generalized to a system with more than three waveguides. The Hamiltonian can be calculated via $H = P^{-1}C + B$ with the propagation constants matrix B, the couplings matrix C, and the power overlap matrix P. With the reasonable assumptions of symmetric coupling and neglecting next-nearest-neighbor couplings together with the necessary condition of symmetric refractive index profile for $\mathcal{PT}$-symmetry the general matrices have the following form,

$$C = \begin{pmatrix} \delta_1 & c_1 & 0 & \cdots & 0 \\ c_1 & \delta_2 & c_2 & \ddots & \vdots \\ 0 & c_2 & \ddots & \ddots & 0 \\ \vdots & \ddots & \ddots & \delta_2 & c_1 \\ 0 & \cdots & 0 & c_1 & \delta_1 \end{pmatrix}, \quad B = \begin{pmatrix} \beta_1 & 0 & \cdots & \cdots & 0 \\ 0 & \beta_2 & \ddots & & \vdots \\ \vdots & \ddots & \ddots & \ddots & \vdots \\ \vdots & & \ddots & \beta_2 & 0 \\ 0 & \cdots & \cdots & 0 & \beta_1 \end{pmatrix}, \quad P = \begin{pmatrix} 1 & \kappa_1 & 0 & \cdots & 0 \\ \kappa_1 & 1 & \kappa_2 & \ddots & \vdots \\ 0 & \kappa_2 & \ddots & \ddots & 0 \\ \vdots & \ddots & \ddots & \ddots & \kappa_1 \\ 0 & \cdots & 0 & \kappa_1 & 1 \end{pmatrix}.$$

After calculating the Hamiltonian, one has to solve the discrete Schrödinger equation (S4) to get the intensity distribution of the single sites. The eigenvalue spectrum of the Hamiltonian yields insights to the different frequency components that determine the dynamics of the system. Of course, as the number of parameters increases with the number of sites, the analytical calculation gets more cumbersome and a numerical calculation may be more appropriate.

Supplementary Note 8: Results of all measurement sets

From two sets of twelve three-waveguide couplers with inter-waveguide spacings ranging from 7 to 18 µm that were fabricated, four sets of measurements were conducted. One set of waveguides was written at a velocity of 100 mm/min (waveguide set 1), the other set at 150 mm/min (waveguide set 2). The injection of light into the structure by a helium-neon laser (633 nm, continuous wave) was performed for both sets by exciting both outer channels consecutively. This results in four measurement sets:

- Measurement set 1: exciting waveguide 1 in waveguide set 1
- Measurement set 2: exciting waveguide 3 in waveguide set 1
- Measurement set 3: exciting waveguide 1 in waveguide set 2
- Measurement set 4: exciting waveguide 3 in waveguide set 2

The numbering of waveguides is according to main text Fig. 4a. While the results of measurement set 1 are presented in main text Fig. 4, the results of the measurement sets 2, 3 and 4 are shown in the following Supplementary Figs. 7-9.

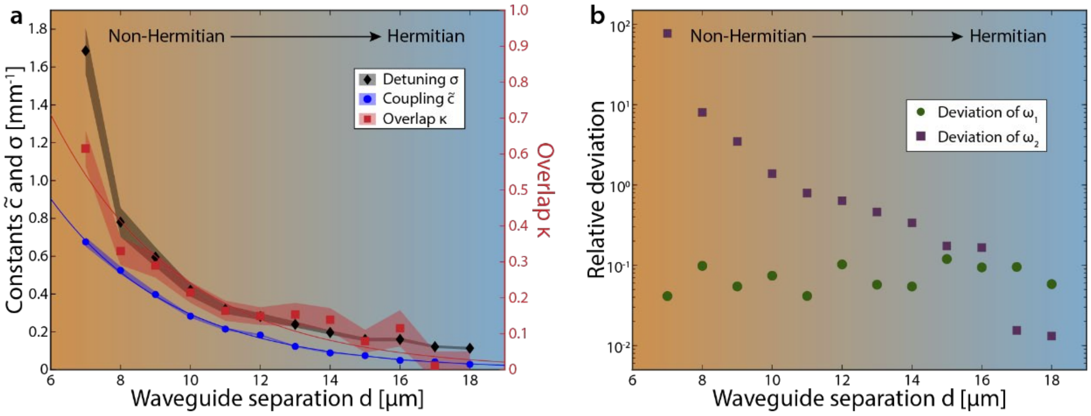

Supplementary Figure 7 | Results of measurement set 2. (**a**) Dependence of coupling $\tilde{c}$, overlap κ and detuning σ on the waveguide separation as determined from best-fit solutions for experiments at separations between 7 and 18 µm spacing. The waveguides were written at a velocity of $100\ \mathrm{mm/min}$ and waveguide 3 was excited. (**b**) In order to construct a reliable indicator for the degree of nonorthogonality present in the system, we compare the relative deviations of the oscillation frequencies ω_1 and ω_2 from their tight-binding limit (i.e. setting $\kappa = 0$). Experiments conducted under conditions where the value of ω_2 exceeds its uncertainty fall within the domain of NOCMT and therefore unequivocally establish non-Hermitian dynamics in the system at hand.

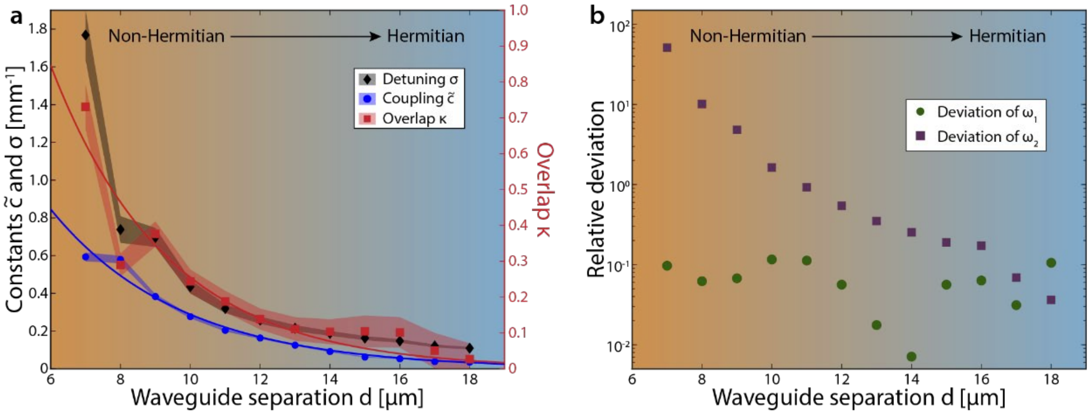

Supplementary Figure 8 | Results of measurement set 3. (**a**) Dependence of coupling $\tilde{c}$, overlap κ and detuning σ on the waveguide separation as determined from best-fit solutions for experiments at separations between 7 and 18 μm spacing. The waveguides were written at a velocity of 150 mm/min and waveguide 1 was excited. (**b**) In order to construct a reliable indicator for the degree of nonorthogonality present in the system, we compare the relative deviations of the oscillation frequencies ω_1 and ω_2 from their tight-binding limit (i.e. setting $\kappa = 0$). Experiments conducted under conditions where the value of ω_2 exceeds its uncertainty fall within the domain of NOCMT and therefore unequivocally establish non-Hermitian dynamics in the system at hand.

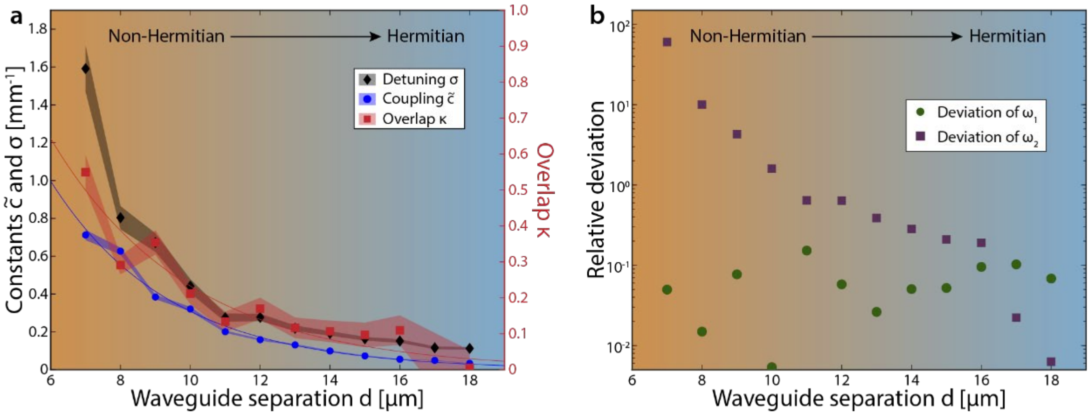

Supplementary Figure 9 | Results of measurement set 4. (**a**) Dependence of coupling $\tilde{c}$, overlap κ and detuning σ on the waveguide separation as determined from best-fit solutions for experiments at separations between 7 and 18 μm spacing. The waveguides were written at a velocity of 150 mm/min and waveguide 3 was excited. (**b**) In order to construct a reliable indicator for the degree of nonorthogonality present in the system, we compare the relative deviations of the oscillation frequencies ω_1 and ω_2 from their tight-binding limit (i.e. setting $\kappa = 0$). Experiments conducted under conditions where the value of ω_2 exceeds its uncertainty fall within the domain of NOCMT and therefore unequivocally establish non-Hermitian dynamics in the system at hand.

Supplementary Note 9: Eigenvalues and -frequencies with and without detuning

This section will deal with the associated problems that may be posed by non-vanishing detuning in the evaluation of the experiment. The influence of the detuning on the system can be investigated theoretically by looking at the two frequencies ω_1 and ω_2 because they determine the dynamics of the system. The frequencies are directly connected to the eigenvalues of the Hamiltonian (see Eq. (S5))

$$\lambda_1 = \tilde{c}\kappa + \omega_1 - \omega_2,$$
$$\lambda_2 = \tilde{c}\kappa,$$
$$\lambda_3 = \tilde{c}\kappa - \omega_1 - \omega_2$$

via

$$\omega_1 = \frac{\lambda_1 - \lambda_3}{2} \quad \text{and} \quad \omega_2 = \frac{2\lambda_2 - \lambda_1 - \lambda_3}{2}.$$

Evidently, the possibility of $\mathcal{PT}$-symmetry breaking is precluded as long as only real couplings are possible, since these eigenvalues cannot be complex in this case.

The eigenstates (supermodes) and their corresponding eigenvalues can be calculated numerically from the refractive index profile of the three-waveguide coupler. The refractive index profile of the waveguides is assumed to be a super-Gaussian of third order (see Supplementary Fig. 10a). In detail, the supermodes $E_1(x,y)$, $E_2(x,y)$ and $E_3(x,y)$ are obtained from the first three eigenfunctions of the 2D paraxial Helmholtz equation (S1) using the split-step Fourier method (SSFM). The eigenvalues are calculated from the normalized eigenstates according to

$$\lambda_i = \int E_i^*(x,y)\left(\frac{1}{2n_0k_0}\left(\partial_x^2 + \partial_y^2\right) + k_0\Delta n(x,y)\right)E_i(x,y)\mathrm{d}x\mathrm{d}y.$$

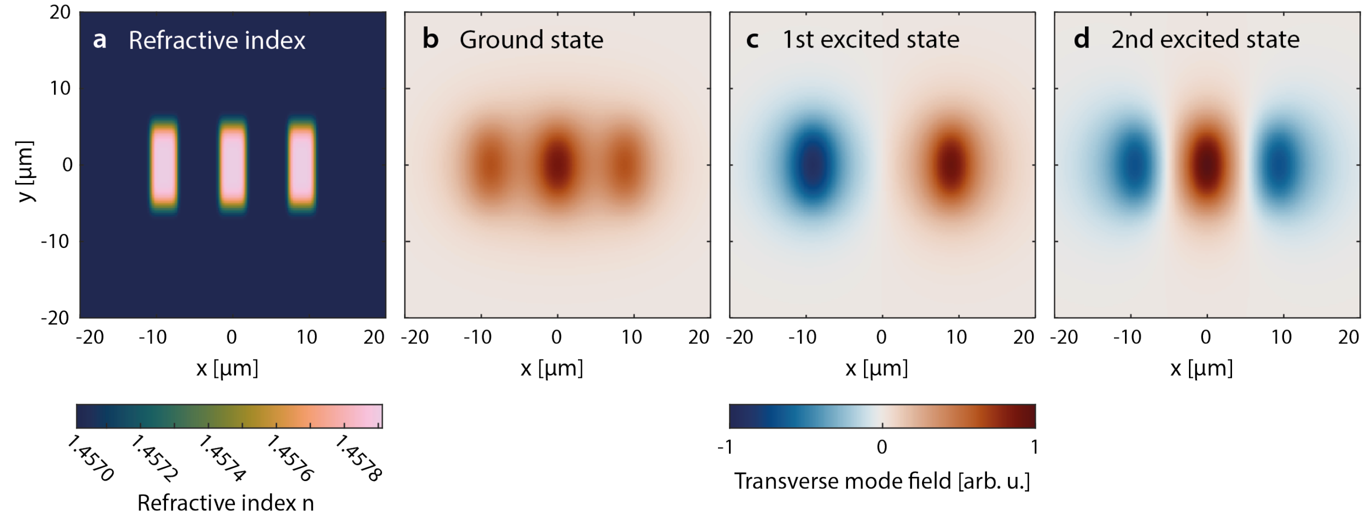

Supplementary Figure 10 | Eigenmodes of the non-detuned three-waveguide coupler with 9 µm separation. (**a**) Refractive index profile. (**b**) Ground state. (**c**) First excited state. (**d**) Second excited state.

The three eigenmodes of the non-detuned three-waveguide coupler are shown in Supplementary Fig. 10b-d for the example of a waveguide separation of 9 μm.

The corresponding eigenfrequencies are shown in Supplementary Fig. 11d,e as function of the waveguide separation. The 1D refractive index profiles for both cases (with and without detuning, respectively) are shown in Supplementary Fig. 11a,b. Supplementary Fig. 11e depicts the detuned case with parameters similar to the main text Fig. 4c, while there is no detuning in Supplementary Fig. 11d. Note that the absolute values of the asymmetry frequency ω_2 is approaching ω_1 at large distances as both parameters are jointly approaching zero:

$$\omega_1(d \to \infty, \sigma > 0) \to \sigma/2 \qquad \text{and} \qquad \omega_2(d \to \infty, \sigma > 0) \to -\sigma/2,$$

which holds true if the coupling $\tilde{c}$ (and the overlap κ) approach zero faster than the detuning σ. In the phase-matched case ($\sigma = 0$), ω_2 falls to zero faster than ω_1 as the coupling frequency depends linearly on the coupling for large distances, while the asymmetry frequency also is linked to the likewise decaying overlap:

$$\omega_1(d \to \infty, \sigma = 0) \to \sqrt{2}\tilde{c} \qquad \text{and} \qquad \omega_2(d \to \infty, \sigma = 0) \to 2\kappa\tilde{c}.$$

Another striking difference between the Supplementary Figs. 11d and 11e is that the asymmetry frequency ω_2 changes its slope at around 10 μm separation if a detuning is present. In case of three identical waveguides, ω_2 is instead monotonically decreasing with a nearly exponential behavior. Both curves can be explained by Eq. (S8). Without detuning, only one of its terms remains, namely $\omega_2 = 2\kappa\tilde{c}$, which obviously is monotonically decreasing (see the curves of $\tilde{c}$ and κ in main text Fig. 4c). For large distances, the detuning is dominating, which is why the asymmetry frequency is negative and approaches zero for large distances similar to the detuning. For smaller distances, the overlap has an increasing influence and ω_2 changes its slope, because the term containing the overlap has a positive sign and is therefore acting opposite to the detuning. The asymmetry frequency may also have a zero at a smaller distance (if $4\tilde{c}\kappa = \sigma$).

Supplementary Fig. 11c shows the experimental results for the detuned system, which closely resemble the simulation in Supplementary Fig. 11e.

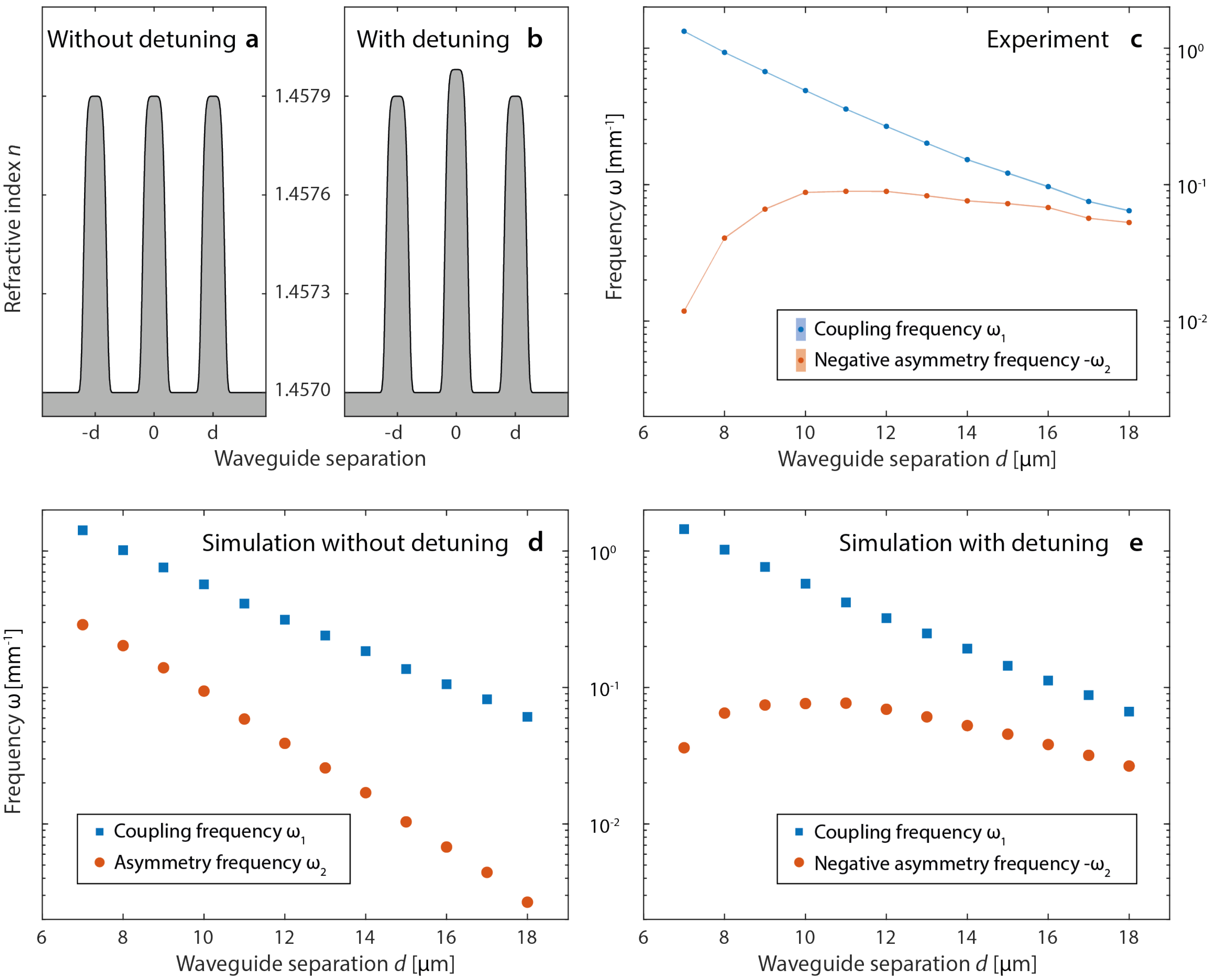

Supplementary Figure 11 | Results for the measured and simulated frequencies of a detuned and non-detuned three-waveguide coupler. (**a**) Simulated frequencies calculated from the first three eigenvalues of the 2D paraxial Helmholtz equation (S1) ignoring detuning. (**b**) Simulated frequencies calculated from the first three eigenvalues of the 2D paraxial Helmholtz equation (S1) considering detuning. (**c**) 1D refractive index profile of a non-detuned three-waveguide coupler with a waveguide separation of d. (**d**) 1D refractive index profile of a detuned three-waveguide coupler. (**e**) Measured coupling and asymmetry frequencies for an experimental setup that has detuning.

Supplementary Note 10: Fluorescence microscopy

Fluorescence microscopy is an established technique for the quantitative observation of intensity dynamics in laser-written waveguides[7,8]. Due to the fabrication process, color centers are exclusively formed in the modified regions serving as waveguide cores. Notably, at the scale of the focal volume (~5 µm, similar to the waveguide diameter), random fluctuations of the color density exist, that show up as a certain level of "graininess" in the full-resolution fluorescence image (Supplementary Fig. 12 at the top). These fluctuations however occur at dimensions several orders of magnitude below the dynamic length scales in our system (determined by the highest beating frequencies observed in the experiment, ~1 mm see Supplementary Fig. 11c). In line with the extremely different aspect ratio of relevant length scales, we reduced the resolution along the direction of propagation by a factor of 55.68, corresponding to an anamorphic pixel size of 6.45 × 6.45 µm. While primarily serving the purpose of avoiding a needless handling of images several tens of thousands of pixels in length, the averaging over 55.68 pixels that goes along with this compression also eliminates virtually all of the fluorescence inhomogeneities (see Supplementary Fig. 12 bottom).

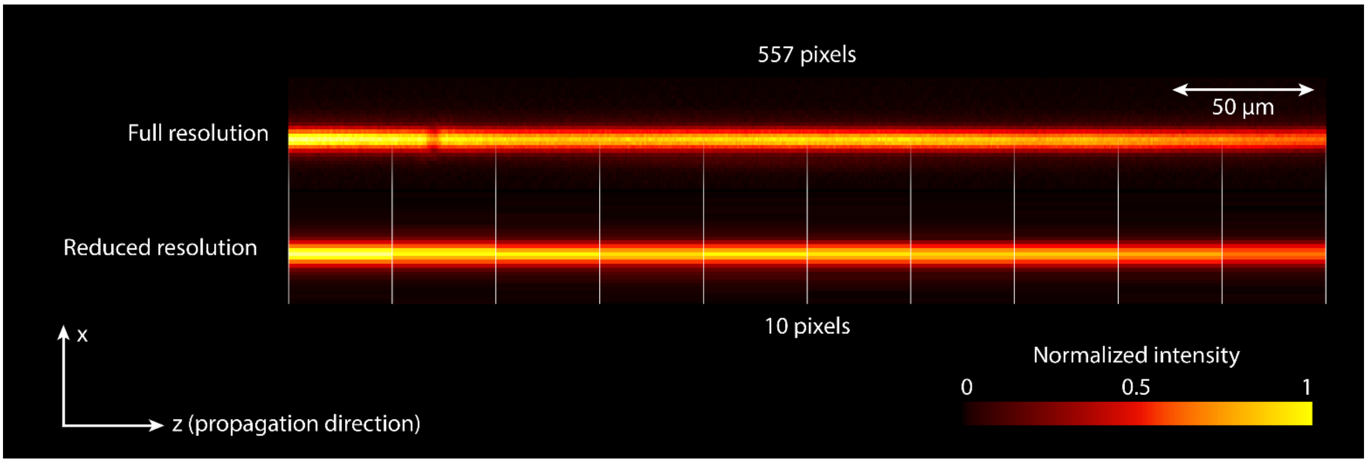

Supplementary Figure 12 | Comparison of fluorescence images of a single waveguide at different resolutions. In the full-resolution fluorescence image (top, pixel size corresponding to 6.45 µm in both x and z direction), certain random fluctuations in the fluorescence efficiency can be observed as "graininess" of the image. However, these occur on or below the scale of the focal volume of the inscription objective (~5 µm). This is 55.68 orders of magnitude below the dynamic length scales in our system, as dictated by the largest coupling coefficients, such that they do not have any impact on the observed physics. Bottom: The reduced lateral resolution eliminates virtually all microfluctuations.

The long-range uniformity of the fluorescence efficiency across the system can be verified by looking at the propagation of a single waveguide. Supplementary Fig. 13 shows that the fluorescence follows the expected exponentially dampened behavior that is due to the constant intrinsic propagation losses of the waveguide. The impact of these propagation losses can be readily separated from the physics of interest by transformation to a co-damped frame.

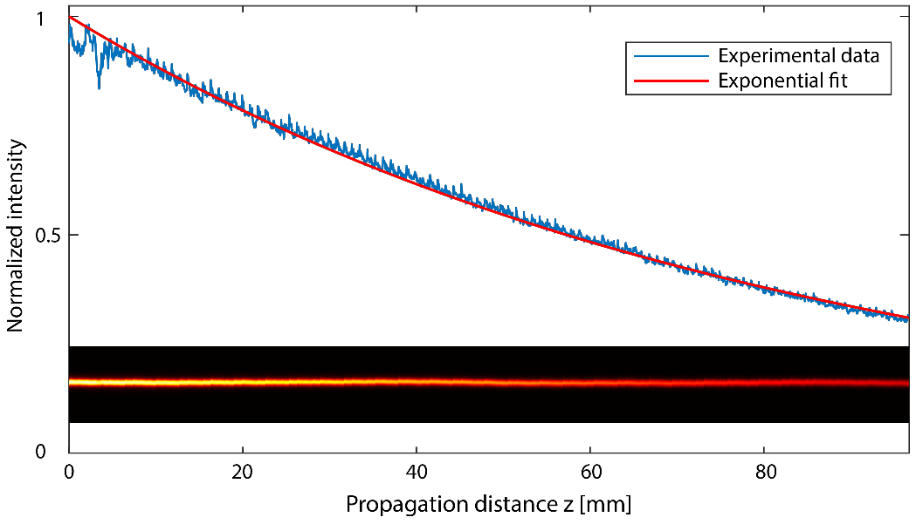

Supplementary Figure 13 | Exponential decay in a single waveguide. The uniformity of the fluorescence efficiency across the sample is demonstrated by observing the expected exponential decay along the propagation direction.

Supplementary References

1. Saleh, B. E. A. & Teich, M. C. *Fundamentals of Photonics*. (Wiley, Hoboken, NJ, 2019).
2. Longhi, S. Quantum-optical analogies using photonic structures. *Laser Photonics Rev.* **3**, 243–261 (2009).
3. Bloch, F. Über die Quantenmechanik der Elektronen in Kristallgittern. *Z. Für Phys.* **52**, 555–600 (1929).
4. Haus, H. A. & Huang, W. Coupled-mode theory. *Proc. IEEE* **79**, 1505–1518 (1991).
5. Mostafazadeh, A. Pseudo-Hermiticity versus PT symmetry: The necessary condition for the reality of the spectrum of a non-Hermitian Hamiltonian. *J. Math. Phys.* **43**, 205–214 (2002).
6. Szameit, A., Dreisow, F., Pertsch, T., Nolte, S. & Tünnermann, A. Control of directional evanescent coupling in fs laser written waveguides. *Opt. Express* **15**, 1579 (2007).
7. Szameit, A. *et al.* Quasi-incoherent propagation in waveguide arrays. *Appl. Phys. Lett.* **90**, 241113 (2007).
8. Szameit, A. & Nolte, S. Discrete optics in femtosecond-laser-written photonic structures. *J. Phys. B At. Mol. Opt. Phys.* **43**, 163001 (2010).
